# Comparative analysis of the chicken IFITM locus by targeted genome sequencing reveals evolution of the locus and positive selection in IFITM1 and IFITM3

**DOI:** 10.1186/s12864-019-5621-5

**Published:** 2019-04-05

**Authors:** Irene Bassano, Swee Hoe Ong, Maximo Sanz-Hernandez, Michal Vinkler, Adebabay Kebede, Olivier Hanotte, Ebele Onuigbo, Mark Fife, Paul Kellam

**Affiliations:** 1 Department of Medicine, Division of Infectious Diseases, Wright Fleming Wing, St Mary’s Campus, Imperial College London, Norfolk Place, London, W2 1PG UK; 2 Wellcome Sanger Institute, Wellcome Genome Campus, Hinxton, Cambridge, CB10 1SA UK; 3 Imperial College London, Department of Life Sciences, South Kensington, London, SW7 2AZ UK; 4 Department of Zoology, Faculty of Science, Charles University, Viničná 7, 128 44 Prague, Czech Republic; 5 Addis Ababa University (AAU), P.O. Box 32853, Addis Ababa, Ethiopia; 6 Amhara Regional Agricultural Research Institute (ARARI), P.O. Box 527, 100 Bahir Dar, Ethiopia; 7 International Livestock Research Institute (ILRI), P. O. Box 5689, Addis Ababa, Ethiopia; 8 School of Life Sciences, University of Nottingham, University Park, Nottingham, NG72RD UK; 9 Center for Tropical Livestock Genetics and Health (CTLGH), The Roslin Institute, University of Edinburgh, Easter Bush, Midlothian, EH25 9RG Scotland, UK; 10 Department of Pharmaceutical Microbiology and Biotechnology, Faculty of Pharmaceutical Sciences, University of Nigeria, Nsukka, Enugu State 410001 Nigeria; 11 The Pirbright Institute, Ash Road, Woking, GU24 0NF UK; 12 Kymab Ltd The Bennet Building (B930) Babraham Research Campus, Cambridge, CB22 3AT UK

**Keywords:** Variant calling, SNPs, INDELs, GATK, Positive selection

## Abstract

**Background:**

The interferon-induced transmembrane (IFITM) protein family comprises a class of restriction factors widely characterised in humans for their potent antiviral activity. Their biological activity is well documented in several animal species, but their genetic variation and biological mechanism is less well understood, particularly in avian species.

**Results:**

Here we report the complete sequence of the domestic chicken *Gallus gallus* IFITM locus from a wide variety of chicken breeds to examine the detailed pattern of genetic variation of the locus on chromosome 5*,* including the flanking genes *ATHL1* and *B4GALNT4*. We have generated chIFITM sequences from commercial breeds (supermarket-derived chicken breasts), indigenous chickens from Nigeria (Nsukka) and Ethiopia, European breeds and inbred chicken lines from the Pirbright Institute, totalling of 206 chickens. Through mapping of genetic variants to the latest chIFITM consensus sequence our data reveal that the chIFITM locus does not show structural variation in the locus across the populations analysed, despite spanning diverse breeds from different geographic locations. However, single nucleotide variants (SNVs) in functionally important regions of the proteins within certain groups of chickens were detected, in particular the European breeds and indigenous birds from Ethiopia and Nigeria. In addition, we also found that two out of four SNVs located in the chIFITM1 (Ser36 and Arg77) and chIFITM3 (Val103) proteins were simultaneously under positive selection.

**Conclusions:**

Together these data suggest that IFITM genetic variation may contribute to the capacities of different chicken populations to resist virus infection.

**Electronic supplementary material:**

The online version of this article (10.1186/s12864-019-5621-5) contains supplementary material, which is available to authorized users.

## Background

The chicken IFITM (chIFITM) locus is clustered on chromosome 5 and contains five genes, of which three are known to be interferon stimulated genes (ISGs) with potent antiviral activity, namely *chIFITM1*, *2, 3* [1, 2]. These proteins restrict viral infections by blocking fusion of the viral and host membranes, thereby interfering with viral entry and replication. It is clear from in vitro studies that genetic variation within IFITM gene sequences can affect the proteins’ activity, their localization and thus the ability to fully restrict viral infections. Indeed, in human IFITM3 (hIFITM3), substitutions at an amino acid residue known to be the core site for binding of the E3 ligase NEDD4 can lead to higher levels of IFITM3 protein abundance by preventing IFITM3 degradation [3]. It was also shown that a synonymous single nucleotide polymorphism (SNP), rs12252-C and rs34481144, in *hIFITM3* gene are associated with increased risk of severe influenza infections [4, 5]. This suggests that as with other ISGs, genetic variation may be linked to within and between species-specific differences in susceptibility to infection or its severity. Although different susceptibility to infection is known in different avian species linked to IFITM variation [2], a systematic IFITM locus survey of genetic variation in different chicken lines and breeds has not been undertaken, due in part to the lack of a reliable chicken genome sequence of this locus.

To date, the study of *IFITM* genetic variation has only been undertaken on a few chicken samples of different origins and no comprehensive breed analysis has been conducted. It is known that chickens within inbred lines display nearly complete homozygosity and thus, only comparison between different inbred lines or with other breeds can shed light on their underlying genetic variation. Elucidation of the chIFITM locus genetic variation can be achieved by comparing different chicken inbred lines that are being used for laboratory screening and kept under SPF (specific-pathogen-free) conditions, commercial chickens and poultry populations kept in geographically-isolated villages in different areas of the world. These do not only represent distinct populations of chickens but also reflect different indigenous pathogen exposures and husbandry considerations such as infection control measures and human selection for productivity and/or likeability traits. We hypothesize that genetic variation across diverse groups of birds will reveal genetic variants with enhanced adaptive antiviral functions.

We have recently showed that targeted pull down of the chIFITM locus can successfully and accurately characterize this locus without the need of performing whole genome sequencing [6]. Here we have used SureSelect probes to pull down the chIFITM locus in a total of 206 chickens, we have assembled the locus using as a reference a Red Jungle Fowl (*Gallus gallus*) we previously sequenced [6] and we performed genetic variation analysis using a modified version of the GATK Best Practice pipeline [7]. Our results catalogue the first comprehensive list of SNVs and INDELs for the chIFITM locus as well as within and between groups of birds variation.

## Methods

### Sample preparation and analysis

A total of 206 chickens were used in this study. We have used SureSelect probes to pull down the chIFITM locus from: 37 European breeds from the Czech Republic, Germany and Slovakia (birds from small farms and hobby breeders), 63 inbred lines (Pirbright Institute), 26 commercial birds (from 8 UK-based supermarkets) and 80 indigenous birds from Nigeria and Ethiopia (Additional file 1: Tables S1 to S4).

DNA was extracted using the Qiagen DNA or Tissue extraction kit according to the manufacturer’s instructions. Up to 5 μg of the extracted DNA were sent to the Wellcome Sanger Institute sequencing facility where the samples were sequenced using Illumina MiSeq sequencers, following targeted SureSelect pull-down of the locus of interest according to the manufacturer’s protocol [6, 8].

Commercial chickens refer to chicken breasts purchased from supermarkets in UK, namely Cambridge, Cambourne and Saffron Walden. Three groups were analysed: standard, free range and organic chicken breast (where available) (Additional file 1: Table S3). European chicken samples (muscle, blood or feathers) were provided by the Genetic bank of the Department of Zoology, Charles University, Prague, Czech Republic. The samples were collected from free-ranging and yarding fowl kept by small farmers and hobby breeders from the Czech Republic, Germany and Slovakia, with one sample per breed for a total number of 37 breeds (Additional file 1: Table S2). The lines belonging to the Pirbright Institute are inbred chickens that have been housed in sterile conditions only for laboratory use (Additional file 1: Table S1). The Nigerian and Ethiopian samples are indigenous chickens from small villages and grown in captivity [9]. The Nigerian indigenous chickens are left to roam in the village, feed from picking earthworms, insects, herbage, household refuse, crop residues, seeds from the ground or in farms and return in the evening to their enclosures. These chickens have never been vaccinated nor treated with antibiotics. Five strains were collected from Nsukka, Nigeria. Each strain represents a genotype. The Ethiopian chickens are native local birds which belong to smallholder farmers from several villages in Addis Ababa, except for Dz-16 and Dz-18 chickens (Additional file 1: Table S4). These two are an indigenous ecotype under a breed improvement program of an agricultural research station performing selection for improved egg and meat yield. None of the Ethiopian chickens have been vaccinated except for the two chickens under selection and similarly all the birds have been fed by free scavenging and occasional left over grain and food supplementation (maize, wheat, rice, teff and sorghum and anything like sands, worms etc.) except for Dz-16 and Dz-18.

Following MiSeq sequencing, FASTQ files were first analysed for low quality reads with FastQC and assembled using as a reference the PacBio consensus reference sequence we previously generated [6]. Mapping and sequence analysis were performed as described in ref. [6]. For variant analysis, the GATK pipeline was used (Genome Analysis Toolkit). In absence of any reliable SNPs/INDELs databases, the analysis was performed as suggested in the Best Practice by the bootstrapping method ([7] GATK Toolkit v3.7). The final list of variants was generated as a VCF file (Variant Call Format) which we were able to visualize using IGV (Integrative genome Visualization tool) from the Broad Institute. SNP densities were calculated using SNiPlay [10].

### Adaptation of the GATK pipeline for analysis of the chicken samples

At the point of this study the NCBI recorded 133 SNP batches submitted since 2002 [11]. These were generated using Samtools and were directed to specific loci, exome or promoter regions; in addition, most of them are mapped to the older version of the chicken genome. None of these batches focused on the *IFITM* genes nor has analysed several chicken breeds, rather one or two types at the time or per study. A complete list of SNPs and INDELs for each of the chicken chromosomes is available from the NCBI website [12].

The GATK pipeline, a well-established protocol for variant analysis, mainly of human samples, was used for our analysis. The GATK pipeline has been successfully adopted for variant discovery on other species, with small modifications to overcome a lack of a well curated SNPs/INDELs databases. For this study, we used the bootstrapping method as suggested by the Best Practice protocol and analysed separately the 37 European, 26 commercial, 63 inbred lines and 80 indigenous chickens. A database of all SNPs/INDELs relative to the reference genome was generated and used to recalibrate the variant calling from the samples. Recalibration graphs were generated for each of the 206 samples with one round of recalibration performed for each sample. The final, filtered SNPs and INDELs were annotated and studied further. Given the number of samples analysed, we refer to single nucleotides variants (SNVs) rather than single nucleotide polymorphisms (SNPs) in our results.

### Positive selection acting on the chIFITM locus

Detection of positive selection on the chIFITM gene sequences was performed separately using the methods FEL (Fixed Effects Likelihood) [13], FUBAR (Fast, Unconstrained Bayesian AppRoximation) [14] and MEME (Mixed Effects Model of Evolution) [15], all accessible from the Datamonkey Adaptive Evolution server [16, 17]. FUBAR was used under the assumption that selection at the chIFITM sites is pervasive throughout the entire phylogeny, whereas MEME assumes selection happened episodically in only subset or subsets of branches. This means MEME is likely to be the more sensitive method and can detect episodic selections that FUBAR misses. FEL is similar to FUBAR except that it was developed to cater to smaller data sets. As we cannot ascertain which assumption is correct, we opted for a consensus approach to interpret the results. Likewise, due to a lack of prior knowledge, we accepted the respective suggested significance levels (cut-off values) for the purpose of discussing the results.

### Structural analysis of the chIFITM proteins

A predicted structure of chIFITM3 was built using the MODELLER package [18]. Secondary structure was predicted by PSIPRED (PSI-blast based secondary structure PREDiction) [19], and the topology built using the previously described human IFITM3 structure [20] as a template. The model of chIFITM3 (43–137) was built after initially removing the disordered N-terminus. The resulting structure was energy minimized and embedded in an explicit DOPC (1,2-Dioleoyl-sn-glycero-3-phosphocholine) membrane. We conducted molecular dynamics as a refinement step, maintaining the secondary structure elements by applying a harmonic restrain on the alpha-helical segments of the model, as described previously for other systems [21]. The AMBER99SB-ILDN force field (from the Assisted Model Building with Energy Refinement” (AMBER) package) [22] was employed, with the Tip3p water model [23]. The simulation was run for 50 ns, using the GROMACS package (GROningen MAchine for Chemical Simulations) [13] under the NPT (isothermal–isobaric) ensemble at 300 K and 1 bar, with temperature coupled under the V-rescale method [24] (every 0.1 ps) and pressure coupled with the semi-isotropic Berendsen method [13] (every 1 ps). The resulting chIFITM3 model was used as a template for chIFITM1 and chIFITM2, which were threaded on the chIFITM3 structure by aligning the secondary structure features. The disordered N-termini were added a posteriori as a random coil for illustration purposes.

### Ethics approval and consent to participate

Ethiopian samples were collected as part of Mr. Abebabay Kebede PhD study, blood samples procedures were approved by and followed International Livestock Research IAUC guidelines (Reference Number IACUC-RC2017–21). Samples were dispatched in the UK following International Guidelines (Nagoya Protocol) and the approval of the Ethiopian Institute of Biodiversity. Czech Republic samples were collected under the approval of the Czech Ministry of Education, Youth and Sports (permits no. 34712/2010–30 and 13,882/2011–30). Nigerian birds handling and experiments were conducted following the guidelines stipulated by University of Nigeria Research Ethics Committee on animal handling and use. Pirbright inbred samples were collected from birds housed at The Pirbright Institute, as authorised under its Home Office Establishment Licence and in accordance with the Code of Practice for the Housing and Care of Animals Bred, Supplied or Used for Scientific Purposes. Birds were euthanized following neck dislocation. The method results in immediate death and is recognized as an approved method under the UK Home Office legislation, Animals (Scientific Procedures) Act 1986.

## Results

### Distribution of SNVs and INDELs across the 40 kb red jungle fowl reference locus

**Fig. 1:**
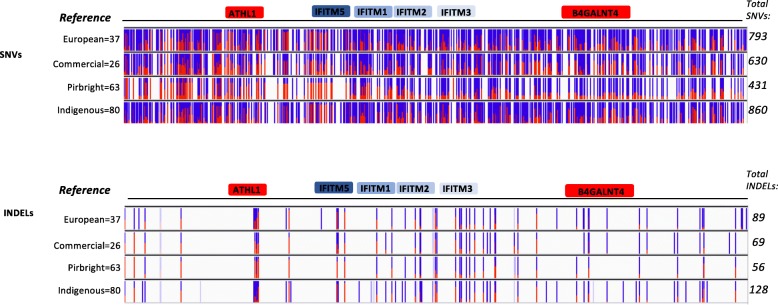
SNVs and INDELS frequency across the 40 kb region. The figure shows the overall distribution of the SNVs and INDELS across the 40 kb reference region. Blue and red bars length indicate the number of samples showing that particular SNVs or INDEL for that position (allele fraction for a single locus). Blue: reference allele, Red: alternate allele. Shaded blue or red indicate filtered entries for that locus in a fraction of the samples. Although many SNVs and INDELs occur cross the *chIFITM* genes, most of these are found in the non-coding regions (genes coding blocks shown). The total number of SNVs found within each group and the total number of samples analyzed per group are indicated (right and left). The PacBio consensus sequence was used as a reference (namely the 40 kb region encompassing the chIFITM locus, including the flanking genes)

**Fig. 2:**
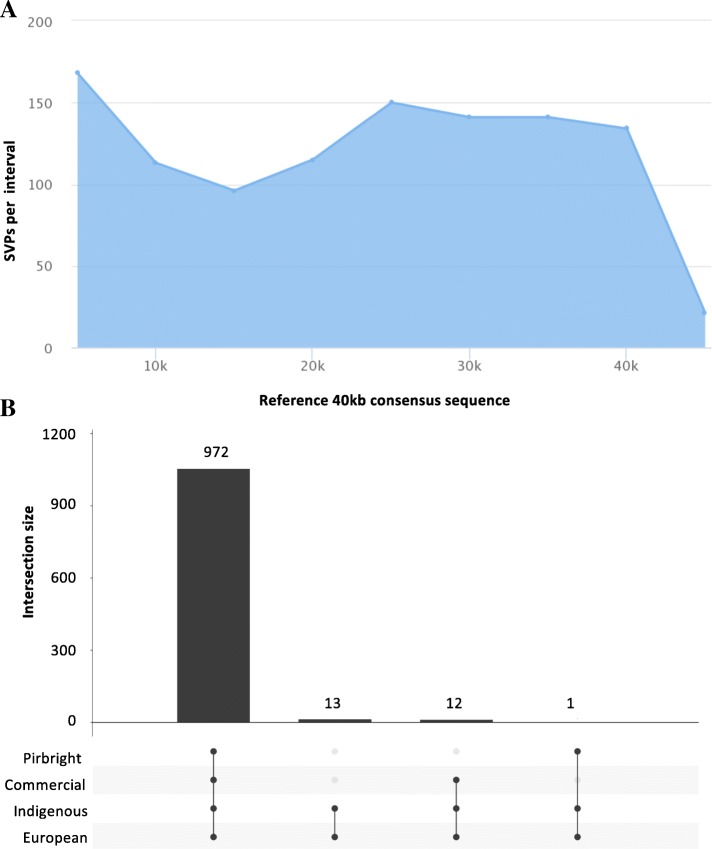
SNV density across the 40 kb Red Jungle Fowl consensus reference sequence. **a** SNP density distribution across the 40Kb region on 5 kb intervals. **b** Common SNPs between the five group of chickens analyzed

Initial analysis across the 40 kb region encompassing the chIFITM locus (including the flanking genes *ATHL1* and *B4GALNT4*) showed the presence of 793, 630, 431 and 860 SNVs for the European, commercial, Pirbright inbred and indigenous chicken samples, respectively, together with 89, 69, 56 and 128 INDELs relative to the reference genome, respectively across the coding and non-coding regions of the locus (Fig. 1). All SNVs are reported in the Variant call file (VCF) Accession number ERP113091. The total number of variants were visualized on IGV by uploading the 40 kb consensus reference sequence we have previously characterized and the VCF files generated by GATK, for each set of the chicken groups. Figure 1 shows the overall SNVs/INDELs distribution expressed as allele fraction for each single locus. We analysed the overall distribution across the 40 kb region in 5 kb intervals as well as the distribution of the SNVs which were common between the four groups of chickens analysed (Fig. 2a-b). In addition to allele frequency, we were able to visualize the genotype within each group (Additional file 1: Figure S1A-D) to investigate the homo-or heterozygosity levels across the samples. This reveals there are a larger number of genetic variants observed in the commercial and village chickens, while the least variation is observed, as expected, in the Pirbright lines. Within this group, most of the SNVs are homozygous for the alternate allele with very few heterozygotes (these samples are colour coded in Additional file 1: Figure S1A-D). Among the groups analysed, a few samples showed limited variation from the reference, namely one commercial line, an Aldi free range chicken sample (Additional file 1: Figure S1B), one of each biological replicates from the Pirbright chicken lines: N, C, P and 6 (Additional file 1: Figure S1C), and nine chicken samples from the Ethiopian and Nigerian group, namely Ibile/wild type, Opipi/featherless, Tssg-07 h, Tssm-68c, Amam-4 h-115, Amam-5c-147 and two Onigbaogbe/Rose chickens (Additional file 1: Figure S1D).

### SNV distribution in the coding regions of the *IFITM1*, *2* and *3* genes

**Fig. 3:**
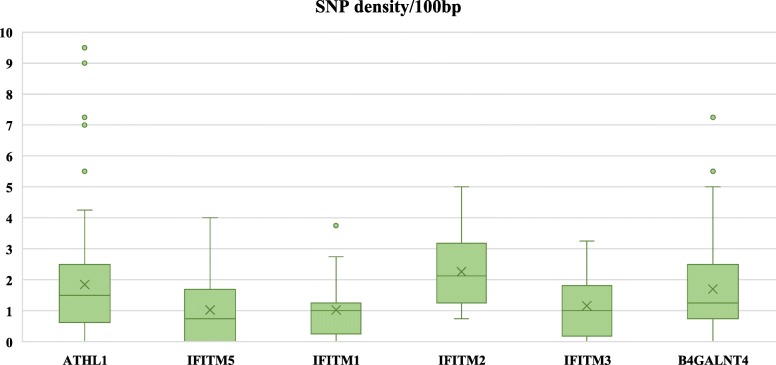
SNV density across the IFITM locus. Average number of SNVs per 100 bp in the *chIFITM* locus, including the flanking genes ATHL1 and B4GALNT4. The latter are 10 kb and 20 kb in length, thus showing more spread data compared to the smaller *chIFITM* genes. Nevertheless, *chIFITM2* shows a higher degree of variability compared to the other IFITM genes

Next, we focused specifically on variant analysis of the *IFITM* genes concentrating on INDELs and SNVs, annotating them within each of the *IFITMs* genes (Table 1, Fig. 3). We analysed separately the four *chIFITMs* genes, focusing only on the coding regions (two exons/IFITM) of the three antiviral *chIFITM1,2,3*. Overall, there were INDELs within the exons for 3 indigenous chicken samples (3 INDELs) in the *chIFITM2* and *chIFITM3* genes, and a total number of 10, 15, 7 and 18 SNVs distributed over the European, commercial, Pirbright and indigenous chicken samples, respectively in their *IFITM* genes (Tables 1 and 2). We calculated the number of SNVs per 100 bp across each of the genes (exons and intron), indicating that *chIFITM2* had the highest number of variants compared to *chIFITM1, 3* and *5* (Fig. 3 and Table 1).

**Table 1:** List of the total number of SNVs and INDELs within each of the *IFITM* genes

	IFITM1	IFITM2	IFITM3	IFITM5	Total/group
SNVs					
European	2	3	2	2	10
Commercial	1	10	2	1	15
Pirbright	–	4	1	1	7
Indigenous	–	10	2	–	18
INDELs					
European	–	–	–	–	–
Commercial	–	–	–	–	–
Pirbright	–	–	–	–	–
Indigenous	–	2	1	–	3

**Table 2:** Annotation of each SNV and INDEL across the *chIFITM1,2,3* genes

	^a^Genomic location	Reference/Variant 3′-5′ (5′-3′)	European	Commercial	Pirbright	Indigenous	Amino acid change (5′-3′)		Codon	Exon
IFITM1	SNVs									
	13,141	G/A (C/T)	√	√	x	x	CGG to TGG	Arg to Trp	77	2
	14,105	C/T (G/A)	√	x	x	x	TCG to TCA	Ser to Ser	56	1
IFITM2	SNVs									
	14,960	G/T (C/A)	√	√	√	√	CGG to AGG	Arg to Arg	111	2
	15,078	G/A (C/T)	x	x	x	√	GGC to GGT	Gly to Gly	72	2
	15,080	C/T (G/A)	x	√	x	√	GGC to AGC	Gly to Ser	72	2
	15,081	T/C (A/G)	√	√	√	√	GTA to GTG	Val to Val	71	2
	15,083	C/T (G/A)	x	√	x	x	GTA to ATA	Val to Ile	71	2
	15,084	G/A (C/T)	x	x	x	√	TTC to TTT	Phe to Phe	70	2
	16,175	A/C (T/G)	√	√	√	√	GCT to GCG	Ala to Ala	54	1
	16,193	G/A (C/T)	x	x	x	√	TTC to TTT	Phe to Phe	48	1
	16,242	T/C (A/G)	x	√	x	√	AAG to AGG	Lys to Arg	32	1
	16,292	G/A (C/T)	x	√	x	x	TAC to TAT	Tyr to Tyr	15	1
	16,295	G/A (C/T)	√	√	√	√	TCC to TCT	Ser to Ser	14	1
	16,303	T/C (A/G)	x	x	x	√	ATG to GTG	Met to Val	12	1
	INDELs									
	16,243–16,244	TG/T (CA/A)	x	x	x	√	Frameshift		31	1
	16,269	A/ACC (T/GGT)	x	x	x	√	Frameshift		23	1
IFITM3	SNVs									
	18,814	T/C (A/G)	√	x	x	x	ATG to GAG	Met to Glu	119	2
	18,862	C/T (G/A)	√	√	√	√	GTC to ATC	Val to Ile	103	2
	19,572	G/A (C/T)	x	x	x	√	TGC to TGA	Cys to STOP	59	1
	INDELs									
	19,576–19,577	AC/A (GT/T)	x	x	x	√	Frameshift		58	1

^a^ These locations refer to the position on the PacBio consensus reference sequence already published and submitted [5]

**Fig. 4:**
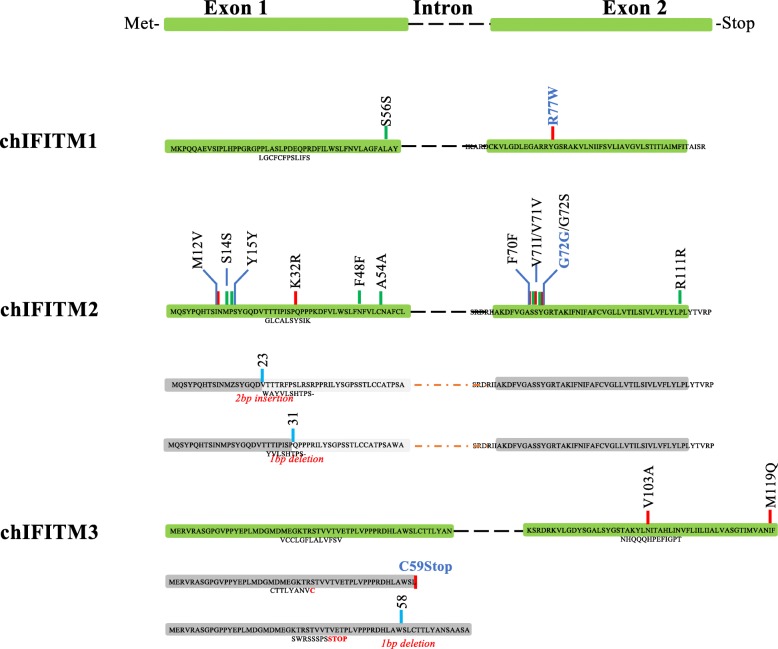
SNVs and INDELS localization across the chIFITM proteins. chIFITM proteins are comprised of two exons separated by one intron. SNVs and INDELS locations from Table 3 are depicted in red (missense SNVs), green (silent SNVs) or in blue (INDELs) bars. An alternate structure is drawn for chIFITM2 and 3 as a result of the frameshift caused by the respective INDELs. Greyed exons represent mutated proteins as a result of the frameshift: in chIFITM2, the two frameshifts in exon 1 generate a novel protein which has lost the IM1 domain; in chIFITM3 the C59STOP generates a shorter protein, with exon 2 completely missing. Aa = amino acid, IM1 = intramembrane domain 1. Bold blue SNVs regarded to be deleterious for the protein as they would disrupt the overall structure (see structural data analysis for G72S and R77W)

For *chIFITM2* there were a total of 12 SNVs among all the groups of chickens of which 6 were new SNVs, not annotated in other studies of chicken variants. These were differently distributed among the chicken groups (Table 2 and Fig. 4): synonymous, R111R, A54A and S14S were found in all the chicken groups with no particular preference for chicken breed or homo/heterozygosity status; synonymous G72G (new) was only observed in 3 of the 80 indigenous chickens (Nigerian chickens Frizzle, Abolorun/Naked and Ibile/Wild type); non-synonymous G72S (new) was found only in 2 of the 26 commercial chickens (Morrison’s and Tesco organic chicken) and 1 of the 80 indigenous chicken (Nigerian chicken Onigbaogbe/Rose); codon 71 of *chIFITM2* had two SNVs: synonymous V71 V and non-synonymous V71I, the latter being found in 2 of the 26 commercial chickens, namely commercial Aldi and Morrisons chicken; while V71 V was observed in all the groups with no particular preference for chickens or breeds. Synonymous F70F (new) was present only in 2 of the 80 indigenous chickens (Ethiopian chickens Amam-3 h-144 and Amam-10 h-108); synonymous F48F was observed only in one of the Nigerian indigenous chicken group, namely the Abolorun/rose group; non-synonymous K32R (new) was found in 2 of the 26 commercial lines (Sainsbury’s free range and Morrisons organic chicken) and one Nigerian sample (Onigbaogbe/rose); synonymous Y15Y (new) was found only in 2 of the 26 commercial samples, namely Aldi and Morrisons; non-synonymous M12 V (new) was observed only in 2 of the 80 indigenous samples, namely the Ethiopian chicken AMAM-3H-124 and AMAM-1H-031. *chIFITM2* also showed two INDELs at codons 23 and 31 respectively resulting in a frameshift altering the length of exon 1 (Table 2 and Fig. 4). Because of this, the conserved IM1 of *chIFITM2* domain is altered and thus it is likely that these INDELs would result in non-functional proteins (Fig. 4). These INDELs were only observed in 1 of the 80 indigenous chickens, Onigbaogbe/Rose (Table 2).

*chIFITM1* and *3* had the least number of variants, with 2 and 3 SNVs each, respectively. *chIFITM1* had two SNVs one in each exon, of which one is synonymous, namely S56S (Table 2 and Fig. 4). S56S was a new SNV within exon one within the IM1 domain. S56S it is detected as a heterozygous SNV and only in 1 of the 37 European chicken samples, namely Plymouth rock. The other SNV in *chIFITM1*, is the non-synonymous R77W, which is heterozygous in 2 of the 37 European chickens (Frizzle and Brahma) and in 10 of the 26 commercial samples and with no particular preference among the various chickens (Table 3 and Fig. 4). This SNV is found in exon two, within the highly conserved CIL domain, and could, therefore, be of functional relevance.

**Table 3:** Positive and negative selection of the chIFITM proteins by FUBAR and MEME

**Codons under episodic positive/diversifying selection**	**FUBAR Prob[a < &b]**	**MEME** ***p*** **-value**
chIFITM1–77	0.961	0.13
chIFITM1–36	0.936	0.16
chIFITM3–103	0.925	0.35
**Codons under episodic negative/purifying selection**	**FUBAR Prob[**a **> &**b**]**	
chIFITM1–7	0.958	
chIFITM1–63	0.921	
chIFITM1–95	0.910	
chIFITM2–54	0.989	
chIFITM2–112	0.988	
chIFITM2–29	0.929	
chIFITM2–14	0.928	
chIFITM2–39	0.928	
chIFITM3–136	0.929	
chIFITM3–134	0.928	
chIFITM3–114	0.927	
chIFITM3–78	0.913	
chIFITM5–58	0.990	
chIFITM5–34	0.924	
chIFITM5–99	0.905	

Values with a threshold of 0.9 (FUBAR) or 0.1 (MEME) were considered

**Fig. 5:**
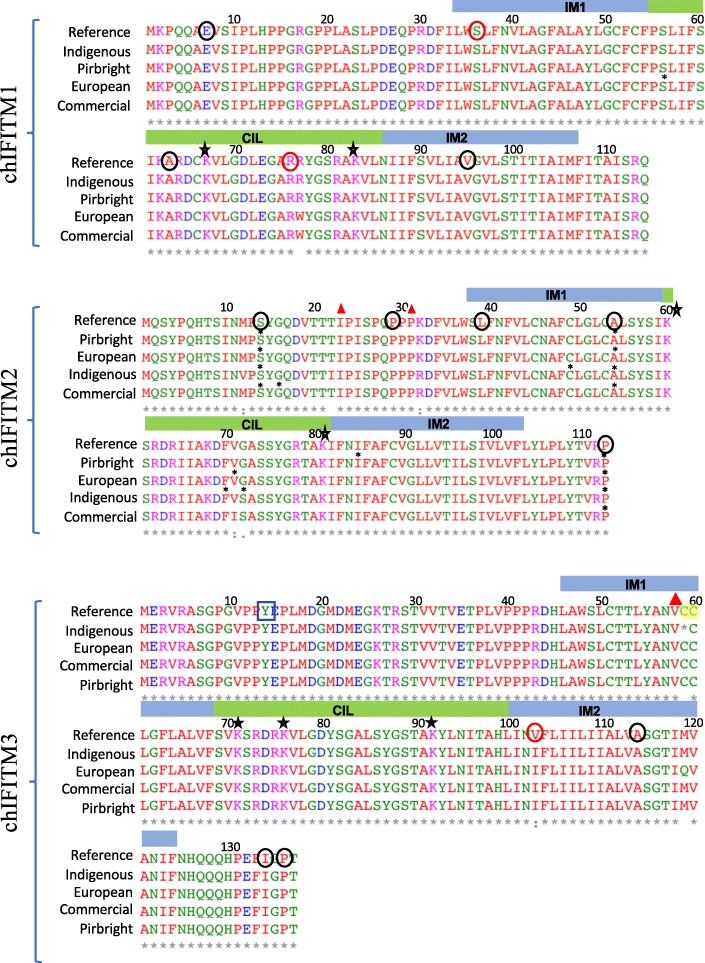
Multiple sequence alignment of the chIFITM proteins showing the mutations across the samples. Amino acid sequence containing the *chIFITM1,2,3* SNVs were aligned using Clustal Omega. IM1/2: intramembrane domain 1 and 2, CIL: conserved intracellular loop, *: silent SNVs. Red triangle: INDELs. Red circles highlight codons under positive selection, black circles codons under negative selection (Refer to Additional file 1: Table S4 for more details), black box: conserved localization signal, star: conserved lysine residues; yellow highlight: conserved double cysteine motif (CC)

*chIFITM3* had three SNVs (all three non-synonymous one of which was a missense mutation), namely M119E, V103I and C59STOP, with the first and last SNVs being new (Table 3 and Fig. 4). The truncated non-sense substitution in *chIFITM3* is expected to be non-functional, missing the full-length exon 2. This mutation was observed only 1 of the 37 European chickens, Maranas where it is heterozygous for the alternate allele. Further, M119E was observed only 1 of the 80 indigenous chickens, Asa/Frizzle. On the other hand, V103I was found in all the chicken groups analysed (both at homo- and heterozygosity) in most of the samples, with no particular preference for chicken groups. We also identified an INDEL in exon 1 of *chIFITM3,* a 1 bp insertion which alters the length of exon 1, effectively elongating exon 1 the coding region but prematurely ending the protein, thus generating a putative non-functional *chIFITM3* (Fig. 4). Interestingly, this INDEL also disrupts the double cysteine residues (CC motif), which is well conserved across the IFITM proteins of several species (Figs. 4 and 5). This insertion was observed in only 1 of the 80 indigenous samples, namely Ko Shamo Bantam.

### Positive selection analysis

**Fig. 6:**
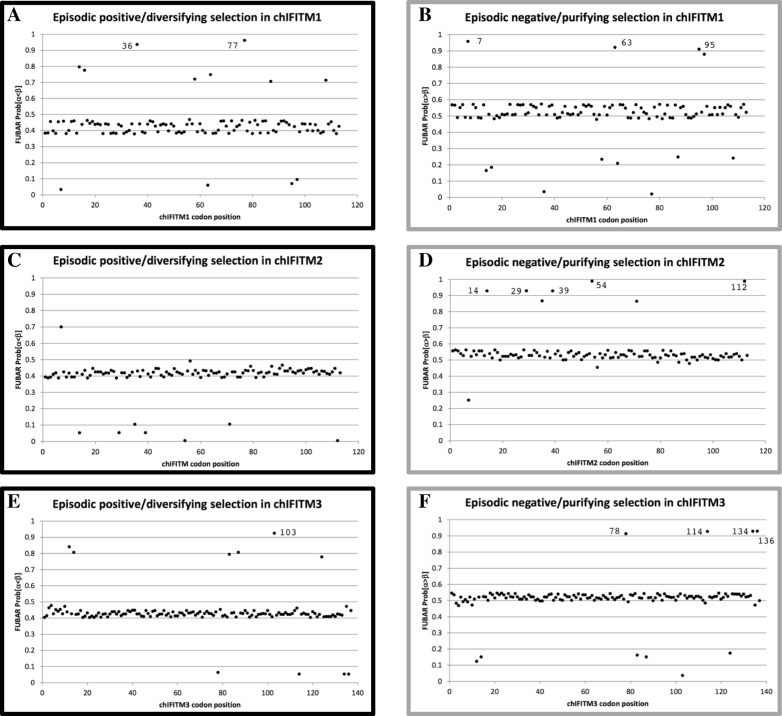
Positive and negative selection analysis of the chIFITM proteins. **a-c-e** Positive selective pressure on chIFITM residues. Residues with Prob[a < b] value over 0.9 is considered as under episodic positive/diversifying selection using FUBAR. **b-d-f** Negative selective pressure on chIFITM residues. Residues with Prob[a > b] value over 0.9 is considered as under episodic negative/purifying selection using FUBAR

Positive selection analysis of the chIFITM locus has been done previously, however it was performed in the context of the whole class Aves which included many other avian species rather than only chickens. It is likely that these results would overestimate selection if considered from the perspective of the chicken lineage [2], in contrast to the analysis reported herein which looks to detect intraspecific selection within the species *Gallus gallus*. As expected, compared to Smith et al., our analyses yielded far fewer positively selected sites. Using the default cut-off value, FUBAR reported the detection of 2 (chIFITM1–36 and chIFITM1–77) and 1 (chIFITM3–103) positively-selected sites in chIFITM1 and chIFITM3, respectively (Fig. 6 and Table 3). ChIFITM1–36 (codon 36) is a Serine in the reference genome and chIFITM1–77 (codon 77) is a Arginine in the reference genome and chIFITM3–103 (codon 103) is an Valine in the reference genome. MEME reported no significant sites in all four chIFITM data sets, although the three FUBAR-reported sites were near to (but did not surpass) the default *p*-value cut-off for MEME (Additional file 1: Figure S2). We also investigated codons that are under negative selection using FUBAR and found a larger number of sites (Table 3). Positive selection detection using the FEL algorithm yielded relatively more hits at p-value threshold of < 0.1 than FUBAR and MEME, but upon closer examination these hits were assessed as most likely to be spurious and therefore not considered further excluded from Table 3 (data not shown).

### Structural analysis of the chIFITM proteins

**Fig. 7:**
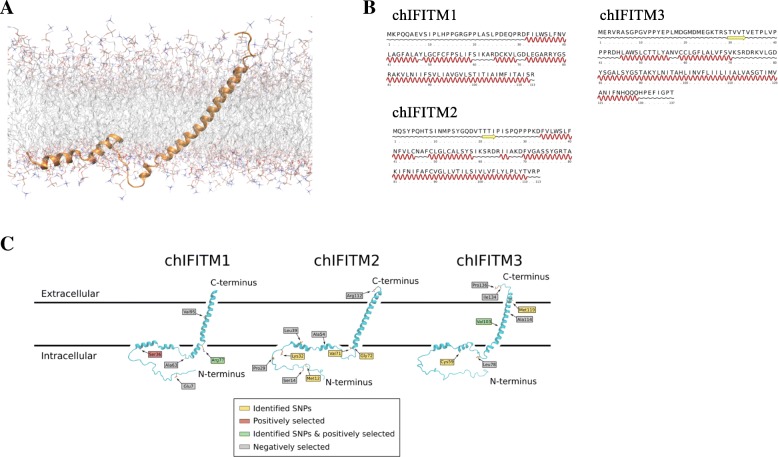
Structural modelling of the chIFITM proteins. **a** chIFITM3 structure model embedded in a DOPC lipid bilayer. The structure was initially modelled based on the published topology of human IFITM3, and later refined by all-atom molecular dynamics. **b** Secondary structure prediction of the three chIFITM proteins according to PSIPRED. All proteins feature an N-terminal disordered domain followed by two short helices and one long C-terminal transmembrane helix. **c** Predicted structure of chIFITM1 and chIFITM2 based on the chFITM3 model. SNVs and positively selected residues identified in this study are highlighted

**Fig. 8:**
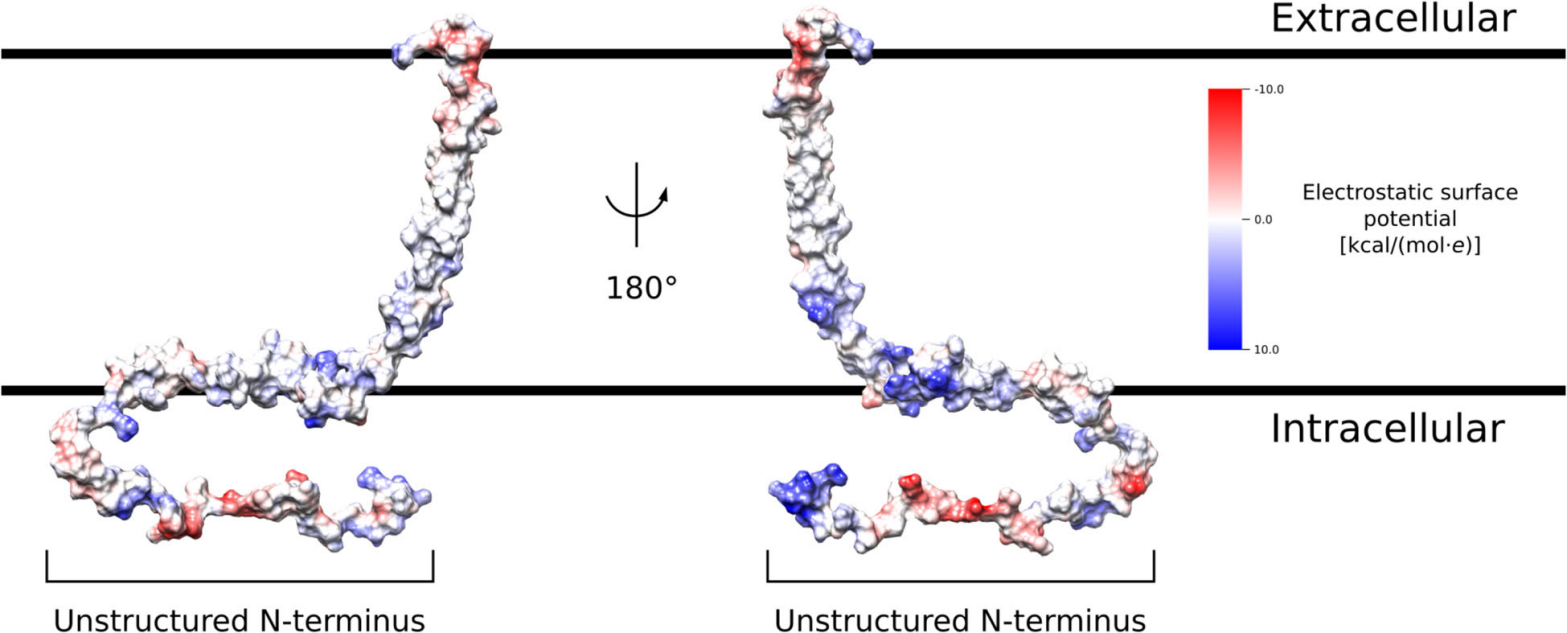
Electrostatic surface potential of the chIFITM3 model. The surface of chIFITM3 is colored according to the Coulombic surface charge of surface accessible residues. The structured segment of the model features a polar (negatively charged) C-terminal cap, a non-polar transmembrane segment and two amphipathic helices on the intracellular face of the membrane. The unstructured N-terminal segment is heavily charged, as expected from a solvent-exposed intrinsically disordered region

The structure of human IFITM3 has been studied and well characterized by NMR [20]. In this study we built a structural model for the avian form of IFITM3 using the human structural information as a template. The membrane topology of the protein was accurately reproduced, with the structure featuring a single transmembrane helix and two amphipathic helices that are adsorbed on the surface of the membrane. The N-terminal amino acids (1–45) are intrinsically unstructured, and thus highly dynamic (Fig. 7). The surface charge of the protein (Fig. 8) further supports this topology. The hydrophobicity of the transmembrane helix is evident, with the N-terminal amino acids (1–45) presenting the typical highly charged nature of disordered protein regions. The helices resting on the surface of the membrane have the amphipathic pattern of hydrophobic residues pointing towards the membrane and polar side-chains exposed to the aqueous solvent. Having an accurate model of the avian chIFITM3 allowed us to use it as a template to also construct models for chIFITM2 and chIFITM1 (Fig. 7c). Secondary structure prediction of all three proteins suggests that they share the same topology, and they also present similar hydrophobicity patterns.

We were able to map the identified SNVs, and positively and negatively selected codons onto our structural models of the proteins (Fig. 7c). In the case of chIFITM2, we found that most SNVs are localized in the intracellular domains, near the boundaries of secondary structure elements. In particular, Gly72 provides the N-terminal break of the transmembrane helix, and the Ser alternative amino acid may lead to a disruption of the overall topology. Further, most negatively selected codons were in the intracellular domain implying its evolutionarily constrained functional or structural significance. In the case of chIFITM1, the positively selected Arg77 is also located at the N-terminal end of the transmembrane helix, on the intracellular surface of the plasma membrane. Being at the bilayer interface, its positively charged sidechains are likely to be involved in protein-membrane contacts with negative phosphate lipid head groups. The removal of that charge, with the non-synonymous SNV A77T leading to a Ile, could therefore lead to an altered membrane topology with putative functional consequences (Fig. 7c). In contrast, *chIFITM3* had negatively selected amino acids and the non-synonymous SNV M199E on the endosomal luminal and cytoplasmic domains. The only non-synonymous and positively selected SNV V103I in contrast is localized on the transmembrane region for chIFITM3. However, no key structural features would be evidently disrupted by this mutation. The SNV for the C59STOP mutation, on the other hand, would result in the removal of the whole transmembrane domain and an inactive protein (Fig. 4).

## Discussion

In this study, we have analysed the genetic variation in the chicken IFITM locus by determining the DNA sequence of the 40kbp locus from 206 chickens, comparing four different chicken groups: European, inbred, commercial and indigenous. We provide an extensive list of SNVs not previously known, mapped to an accurate reference genome, allowing easy re-mapping to other chicken genomes available on NCBI [12]. Our analysis shows genetic variation not only between the groups of geographically distant chickens, but also within groups of related chickens, identifying genetic diversity in this important anti-viral gene locus that may affect the ability to restrict different avian viruses.

Based on our analysis, *chIFITM2* appears to be the most variable gene compared to *chIFITM1* and *chIFITM3,* although the total number of variants was generally small with 17 SNVs and 3 INDELs identified across the 206 samples for *chIFTM1–3* genes (exons only) (Table 2, Fig. 3). We found a total of 12 SNVs in *chIFITM2,* 6 of which were new and two INDELs, both new. Interestingly, 8 of the SNVs were synonymous/silent substitutions, thus presumably not altering the function of the protein. However, two non-synonymous SNVs in the CIL (Conserved Intracellular Loop) domain and two at the N terminus, before the IM1 (Intra-Membrane 1) domain (Figs. 4, 6 and Table 2) were found in 10 chickens (5 commercial and three indigenous), none of which were homozygous. Of note the non-synonymous SNVs at codon 32 of commercial and indigenous chickens changes one of the lysine residues regarded to be a conserved codon important for regulation of protein turnover via ubiquitination [25]. Both INDELs, result in frameshifts predicted to lead to truncated, non-functional proteins and occurred as rare events in 2 indigenous chickens.

Similarly, *chIFITM1* showed a non-synonymous SNV in the CIL domain (R77W), described as conserved by others [26]. The fact that this SNV occurs in heterozygote form in 39% (10/26) of the samples from commercial birds but no homozygotes were observed suggests that this mutation maybe deleterious. Together our analysis suggests that rare deleterious mutations occur in *chIFITM2–3* genes in chickens with some occurring (R77W in *chIFITM1*) at high allele frequency in commercial breeds. Whether these affect the susceptibility of some birds to viral infections or alter the severity of such infections should be tested and could be molecular targets for breeding programmes.

When considering evidence for positive and negative selection across the *chIFTM1–3* genes we observed that residues under negative selection predominate and are often located on the intracellular N-terminus or extracellular/intra-endosomal C-terminus. Rare positively selected codons were identified mostly (two out of three) at the intracellular N-terminus. The predominance of negative selection may reflect a skewed chicken sampling where 42% (89/211) samples were from commercial or inbred chickens. Alternatively, our analysis may support the view that modern chickens have lost a high number of alleles in a relatively short period of time from their genomes compared to the nineteenth century breeds, which applies also to the important anti-viral genes studied here [27]. We suggest therefore, that the commercial-scale breeding of the chicken, does not provide a strong positive selection pressure acting on the three short *chIFITM* genes for anti-viral traits. Larger studies focusing on non-inbred chickens will be required to identify more relevant genetic diversity relating to infection by viruses.

## Conclusions

Here we have generated the first time a list of true, high confidence SNVs and INDELs for the chIFITM locus. Our results show that of the three *IFITMs*, *chIFITM2* contains the highest number of SNVs, compared to *chIFITM1,* and *chIFITM3* shows the least. Positive and negative selection analysis coupled with structure-based mapping of amino acidic changes shows most genetic variation occurs in the unstructured N-terminal cytoplasmic domain suggesting a functional significance. Furthermore, detailed experimental assignment of infection restricting and promoting phenotypes of the amino acids under selection or the observed other non-synonymous variants is now needed to determine if any confer traits of importance for breeding.

## Additional file

Additional file 1: **Table S1.** List of the inbred lines from the Pirbright Institute (Adapted from http://www.narf.ac.uk/chickens/lines.html). The table shows a list of all the inbred lines from the Pirbright Institute together with relevant information regarding each line. ENA ID is provided for each group. **Table S2.** List of the European chicken breeds. The table shows a list of all the European breeds and their origin (muscle or blood). ENA ID is provided for each group. **Table S3.** List of the commercial chickens purchased across UK. The table shows a list of all the supermarket-derived chicken breast purchased between Cambridge, Saffron Walden and Cambourne in 2016. Chickens were also classified based on their origin: standard, free range or organic. ENA ID is provided for each group. **Table S4.** List of the indigenous chickens from Nigeria and Ethiopia. The table shows a list of all the indigenous chickens from Nigeria and Ethiopia. Additional information regarding the village of origin is shown. ENA ID is provided for each group. **Figure S1.** IGV SNVs view and density across the 40Kb region. The VCF file generated by GATK was uploaded using IGV. The figure shows a snapshot of the full-length locus, including the flanking genes ATHL1 and B4GALNT4. Samples showing high levels of heterozygosity are highlighted on the right side of the figure. Blue: SNVs heterozygous for the alternate allele, cyan: SNVs homozygous for the alternate allele, grey: SNVs homozygous for the reference allele, white: no call from GATK. A.: European breeds, B.: commercial chickens from UK supermarkets. C.: inbred lines from the Pirbright Institute, D.: indigenous chickens from Ethiopia and Nigeria. Refer to **Tables S1-S4** for additional for information about the single samples. (PDF 5254 kb)

## Abbreviations

AMBER99SB-ILDN: Assisted Model Building with Energy Refinement” (AMBER) package
chIFITM: Chicken interferon-induced transmembrane
CIL: Conserved Intracellular Loop
DOPC: 1,2-Dioleoyl-sn-glycero-3-phosphocholine
FEL: Fixed Effects Likelihood
FUBAR: Fast, Unconstrained Bayesian AppRoximation
GATK: Genome Analysis Toolkit
GROMACS: GROningen MAchine for Chemical Simulations
hIFITM: Human interferon-induced transmembrane
IGV: Integrative genome Visualization
IM1, 2: Intra-Membrane 1 and 2
INDELs: Insertions/deletions
ISGs: Interferon stimulated genes
MEME: Mixed Effects Model of Evolution
NPT: Isothermal–isobaric
PSIPRED: PSI-blast based secondary structure PREDiction
SNPs: Single nucleotide polymorphisms
SNVs: Single nucleotide variants
VCF: Variant Call Format
