# Supplementary material for: Comparative analysis of the chicken IFITM locus by targeted genome sequencing reveals evolution of the locus and positive selection in IFITM1 and IFITM3
Source: BMC Genomics. 2019 Apr 5;20:272. doi: 10.1186/s12864-019-5621-5 (PMC6451222; doi:10.1186/s12864-019-5621-5)
Supplement: Supplementary file 1 — Table S1. List of the inbred lines from the Pirbright Institute (Adapted from http://www.narf.ac.uk/chickens/lines.html). The table shows a list of all the inbred lines from the Pirbright Institute together with relevant information regarding each line. ENA ID is provided for each group. Table S2. List of the European chicken breeds. The table shows a list of all the European breeds and their origin (muscle or blood). ENA ID is provided for each group. Table S3. List of the commercial chickens purchased across UK. The table shows a list of all the supermarket-derived chicken breast purchased between Cambridge, Saffron Walden and Cambourne in 2016. Chickens were also classified based on their origin: standard, free range or organic. ENA ID is provided for each group. Table S4. List of the indigenous chickens from Nigeria and Ethiopia. The table shows a list of all the indigenous chickens from Nigeria and Ethiopia. Additional information regarding the village of origin is shown. ENA ID is provided for each group. Figure S1. IGV SNVs view and density across the 40Kb region. The VCF file generated by GATK was uploaded using IGV. The figure shows a snapshot of the full-length locus, including the flanking genes ATHL1 and B4GALNT4. Samples showing high levels of heterozygosity are highlighted on the right side of the figure. Blue: SNVs heterozygous for the alternate allele, cyan: SNVs homozygous for the alternate allele, grey: SNVs homozygous for the reference allele, white: no call from GATK. A.: European breeds, B.: commercial chickens from UK supermarkets. C.: inbred lines from the Pirbright Institute, D.: indigenous chickens from Ethiopia and Nigeria. Refer to Tables S1-S4 for additional for information about the single samples. (PDF 5254 kb) [file 12864_2019_5621_MOESM1_ESM.pdf]

	Line N	Line C	Line P	Line 6	Line 0	Line 7	Line 15I
Individuals	9	10	9	9	9	8	10
Breed	White Leghorn	White Leghorn	White Leghorn	White Leghorn	White Leghorn	White Leghorn	White Leghorn
Origin	Cornell University	Reaseheat Laboratory	Cornell University	ADOL East Lansing, 1939	ADOL, East Lansing	ADOL East Lansing, 1939	ADOL East Lansing, 1939
Imported	1982	1969	1996	1972	1985	1972	1962
MHC	B^{21}	B^{4}/B^{4}, B^{12}/B^{12}, B^{4}/B^{13}	B^{19}	B^{2}	B^{21}	B^{2}	B^{15}
Susceptible		LLV Subgroup A, B, C, D	MDV	LLV Subgroup A, B, C, D	LLV Subgroup A, B, C, D	LLV Subgroup A, B, C, D	LLV subgroups A, C; segregating for B, D, E. Moderately susceptible to MDV
Resistant	MDV	MDV		MDV, LLV tumor development	MDV	MDV, LLV tumor development	
Type	Partial Inbred	Inbred	Partial Inbred	Inbred	Partial Inbred	Inbred	
Histocompatible				Line 7_2 skin grafting, cell exchange		Line 6_1 skin grafting, cell exchange	
ENA sample ID	20172_1#1<br>20172_1#9<br>20172_1#17<br>20172_1#25<br>20172_1#33<br>20172_1#39<br>20172_1#46<br>20172_1#53<br>20172_1#60	20172_1#2<br>20172_1#10<br>20172_1#18<br>20172_1#26<br>20172_1#34<br>20172_1#40<br>20172_1#47<br>20172_1#54<br>20172_1#61<br>20172_1#65	20172_1#3<br>20172_1#11<br>20172_1#19<br>20172_1#27<br>20172_1#35<br>20172_1#41<br>20172_1#48<br>20172_1#55<br>20172_1#66	20172_1#4<br>20172_1#12<br>20172_1#20<br>20172_1#28<br>20172_1#36<br>20172_1#42<br>20172_1#49<br>20172_1#56<br>20172_1#62	20172_1#5<br>20172_1#13<br>20172_1#21<br>20172_1#29<br>20172_1#43<br>20172_1#50<br>20172_1#57<br>20172_1#63<br>20172_1#67	20172_1#6<br>20172_1#14<br>20172_1#22<br>20172_1#30<br>20172_1#37<br>20172_1#44<br>20172_1#51<br>20172_1#58	20172_1#7<br>20172_1#15<br>20172_1#23<br>20172_1#31<br>20172_1#38<br>20172_1#45<br>20172_1#52<br>20172_1#59<br>20172_1#64<br>20172_1#68

Total: 63

ENA ID	Breed	Tissue type
20819_1#6	Brakel	Muscle
20819_1#7	Frizzle	Muscle
20819_1#8	La Fléche	Muscle
20819_1#9	Minorca	Muscle
20819_1#10	Ko Shamo Bantam	Muscle
20819_1#11	Minorca	Muscle
20819_1#12	Yokoama	Muscle
20819_1#13	Booted Bantam	Muscle
20819_1#14	Brahma	Muscle
20819_1#15	Czeck	Muscle
20819_1#16	Game Shamo	Muscle
20819_1#17	Japanese Bantam	Muscle
20819_1#18	Padua Bantam	Muscle
20819_1#19	Pekin Bantam	Muscle
20819_1#20	Wyandotte	Muscle
20819_1#21	Plymouth Rock	Blood
20819_1#22	Leghorn	Blood
20819_1#23	Amrock	Blood
20819_1#25	Ko Shamo Bantam	Blood
20819_1#26	Asil	Blood
20819_1#27	Rhode-Island	Blood
20819_1#28	Australop	Blood
20819_1#29	Vorwerk	Blood
20819_1#30	Marans	Blood
20819_1#31	Barbu D'Anvers	Blood
20819_1#32	Czech bt.	Blood
20819_1#33	Barnevelder Bantam	Muscle
20819_1#35	K71 Leghorn	Muscle
20819_1#36	Silkie	Muscle
20819_1#37	Araucana	Muscle
20819_1#38	Barnevelder	Muscle
20819_1#39	Padua	Muscle
20819_1#40	Phoenix	Muscle
20819_1#41	Rosecomb Bantam	Muscle
20819_1#42	Sebright Bantam	Muscle
20819_1#43	Transylvanian Naked Neck	Muscle
20819_1#44	Welsummer	Muscle

Total: 37

Supermarket	Standard	Free range	Organic	Tissue type
	ENA ID			
**Aldi**	20172_1#69	20819_1#57	NA	Breast
**Cooperative**	20172_1#16<br>20172_1#70<br>20819_1#5	20819_1#46	NA	Breast
**M&S**	20172_1#24<br>20172_1#71	20819_1#58	20819_1#59	Thighs/Breast
**Sainsbury's**	20172_1#32<br>20172_1#72<br>20819_1#2	20819_1#50	20819_1#49	Breast
**Tesco**	20819_1#1	20819_1#52	20819_1#51	Breast
**Waitrose**	20172_1#74<br>20819_1#34	20819_1#60	20819_1#53	Breast
**Morrison's**	20819_1#47	NA	20819_1#48	Breast
**ASDA**	20819_1#45	20819_1#56	NA	Breast

Total: 26

ENA ID	Sample	Provenience/village	Breed	Sample	ENA ID	Provenience	Breed
24083_1#1	Abb-3c	Ethiopia/Batambie	NA	11	24083_1#42	Nigeria	Onigbaogbe/Rose comb
24083_1#2	Abb-4h2	Ethiopia/Batambie	NA	12	24083_1#43	Nigeria	Onigbaogbe/Rose comb
24083_1#3	Afa-6h	Ethiopia/Amesha Shinkuri	NA	13	24083_1#44	Nigeria	Onigbaogbe/Rose comb
24083_1#4	Asa-4c	Ethiopia/Ashuda	NA	14	24083_1#45	Nigeria	Onigbaogbe/Rose comb
24083_1#5	Asa-8c	Ethiopia/Ashuda	NA	15	24083_1#46	Nigeria	Onigbaogbe/Rose comb
24083_1#6	Asa-9c	Ethiopia/Ashuda	NA	16	24083_1#47	Nigeria	Onigbaogbe/Rose comb
24083_1#7	Asd-2h	Ethiopia/Dikuli	NA	17	24083_1#48	Nigeria	Onigbaogbe/Rose comb
24083_1#8	Dz-18	Ethiopia/Improved Horro	NA	18	24083_1#49	Nigeria	Onigbaogbe/Rose comb
24083_1#9	Dz-26	Ethiopia/Improved Horro	NA	19	24083_1#50	Nigeria	Onigbaogbe/Rose comb
24083_1#10	Agt-3h	Ethiopia/Tsion Teguaz	NA	20	24083_1#51	Nigeria	Onigbaogbe/Rose comb
24083_1#11	Agt-6h	Ethiopia/Tsion Teguaz	NA	21	24083_1#52	Nigeria	Abolorun/Naked neck
24083_1#12	Tssm-64h	Ethiopia/Metkilimat	NA	22	24083_1#53	Nigeria	Abolorun/Naked neck
24083_1#13	Tssm-68c	Ethiopia/Metkilimat	NA	23	24083_1#54	Nigeria	Abolorun/Naked neck
24083_1#14	Tssg-07h	Ethiopia/Metkilimat	NA	24	24083_1#55	Nigeria	Abolorun/Naked neck
24083_1#15	Afdk-c3	Ethiopia/Kefis	NA	25	24083_1#56	Nigeria	Abolorun/Naked neck
24083_1#16	Amna-7h-144	Ethiopia/Negasi Amba	NA	26	24083_1#57	Nigeria	Abolorun/Naked neck
24083_1#17	Amam-3h-124	Ethiopia/Alfa Midir	NA	27	24083_1#58	Nigeria	Abolorun/Naked neck
24083_1#18	Amam-4h-115	Ethiopia/Alfa Midir	NA	28	24083_1#59	Nigeria	Abolorun/Naked neck
24083_1#19	Amam-5c-147	Ethiopia/Alfa Midir	NA	29	24083_1#60	Nigeria	Abolorun/Naked neck
24083_1#20	Amam-6h-120	Ethiopia/Alfa Midir	NA	30	24083_1#61	Nigeria	Abolorun/Naked neck
24083_1#21	Amam-7c-116	Ethiopia/Alfa Midir	NA	31	24083_1#62	Nigeria	Opipi/featherless wing
24083_1#22	Amam-9c-107	Ethiopia/Alfa Midir	NA	32	24083_1#63	Nigeria	Opipi/featherless wing
24083_1#23	Amam-10h-108	Ethiopia/Alfa Midir	NA	33	24083_1#64	Nigeria	Opipi/featherless wing
24083_1#24	Amam-1h-031	Ethiopia/Alfa Midir	NA	34	24082_1#1	Nigeria	Opipi/featherless wing
24083_1#25	Amam-2c-151	Ethiopia/Alfa Midir	NA	35	24082_1#2	Nigeria	Opipi/featherless wing
24083_1#26	Odb-1h-076	Ethiopia/Bekele Girissa	NA	36	24082_1#3	Nigeria	Opipi/featherless wing
24083_1#27	Odb-8h-013	Ethiopia/Bekele Girissa	NA	37	24082_1#4	Nigeria	Opipi/featherless wing
24083_1#28	Ods-5c-086	Ethiopia/Bekele Girissa	NA	38	24082_1#5	Nigeria	Opipi/featherless wing
24083_1#29	Sdl-3c-019	Ethiopia/Loya	NA	39	24082_1#6	Nigeria	Opipi/featherless wing
24083_1#30	Sdl-6h-057	Ethiopia/Loya	NA	40	24082_1#7	Nigeria	Opipi/featherless wing
24083_1#31	Sdl-8c-090	Ethiopia/Loya	NA	41	24082_1#8	Nigeria	Ibile/Wild type
24083_1#32	1	NIgeria	Asa/Frizzle feather	42	24082_1#9	Nigeria	Ibile/Wild type
24083_1#33	2	Nigeria	Asa/Frizzle feather	43	24082_1#10	Nigeria	Ibile/Wild type
24083_1#34	3	Nigeria	Asa/Frizzle feather	44	24082_1#11	Nigeria	Ibile/Wild type
24083_1#35	4	Nigeria	Asa/Frizzle feather	45	24082_1#12	Nigeria	Ibile/Wild type
24083_1#36	5	Nigeria	Asa/Frizzle feather	46	24082_1#13	Nigeria	Ibile/Wild type
24083_1#37	6	Nigeria	Asa/Frizzle feather	47	24082_1#14	Nigeria	Ibile/Wild type
24083_1#38	7	Nigeria	Asa/Frizzle feather	48	24082_1#15	Nigeria	Ibile/Wild type
24083_1#39	8	Nigeria	Asa/Frizzle feather	49	24082_1#16	Nigeria	Ibile/Wild type
24083_1#40	9	Nigeria	Asa/Frizzle feather	50	24082_1#17	Nigeria	Ibile/Wild type
24083_1#41	10	Nigeria	Asa/Frizzle feather				

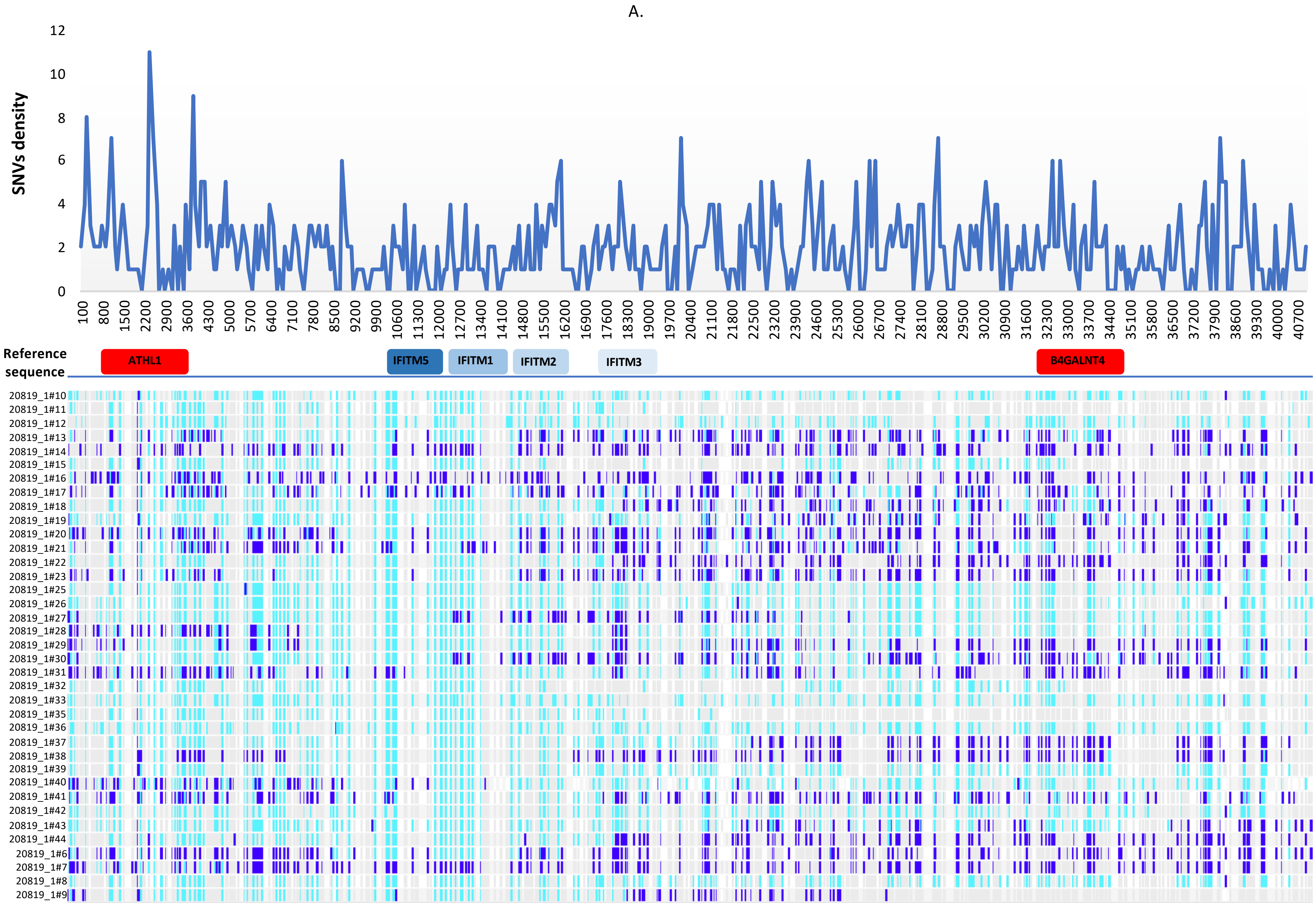
A.
SNVs density
Reference sequence
ATHL1
IFITM5
IFITM1
IFITM2
IFITM3
B4GALNT4
20819_1#10
20819_1#11
20819_1#12
20819_1#13
20819_1#14
20819_1#15
20819_1#16
20819_1#17
20819_1#18
20819_1#19
20819_1#20
20819_1#21
20819_1#22
20819_1#23
20819_1#25
20819_1#26
20819_1#27
20819_1#28
20819_1#29
20819_1#30
20819_1#31
20819_1#32
20819_1#33
20819_1#35
20819_1#36
20819_1#37
20819_1#38
20819_1#39
20819_1#40
20819_1#41
20819_1#42
20819_1#43
20819_1#44
20819_1#6
20819_1#7
20819_1#8
20819_1#9

B.

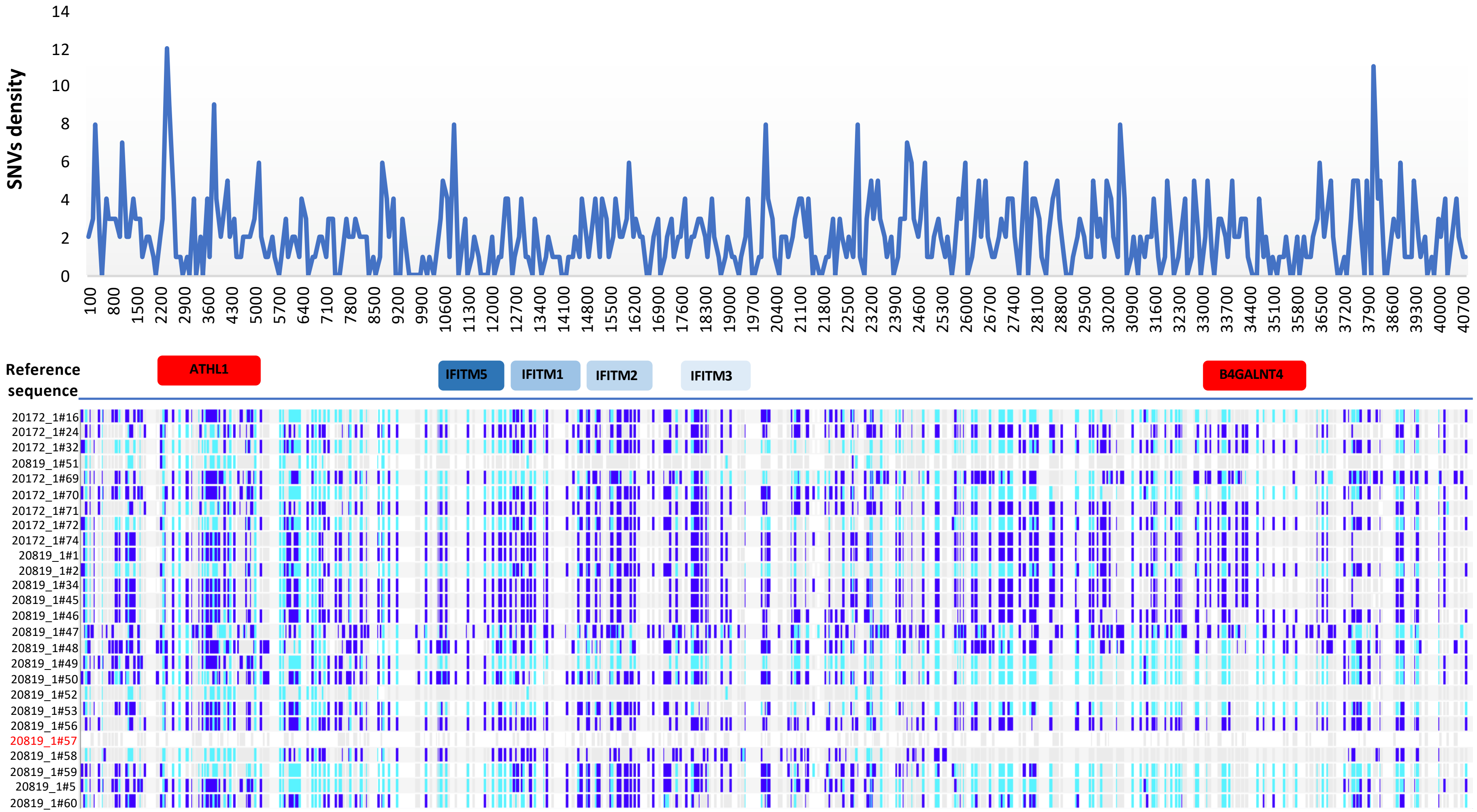
SNVs density
14
12
10
8
6
4
2
0
Reference sequence
ATHL1
IFITM5
IFITM1
IFITM2
IFITM3
B4GALNT4
20172_1#16
20172_1#24
20172_1#32
20819_1#51
20172_1#69
20172_1#70
20172_1#71
20172_1#72
20172_1#74
20819_1#1
20819_1#2
20819_1#34
20819_1#45
20819_1#46
20819_1#47
20819_1#48
20819_1#49
20819_1#50
20819_1#52
20819_1#53
20819_1#56
20819_1#57
20819_1#58
20819_1#59
20819_1#5
20819_1#60

C.

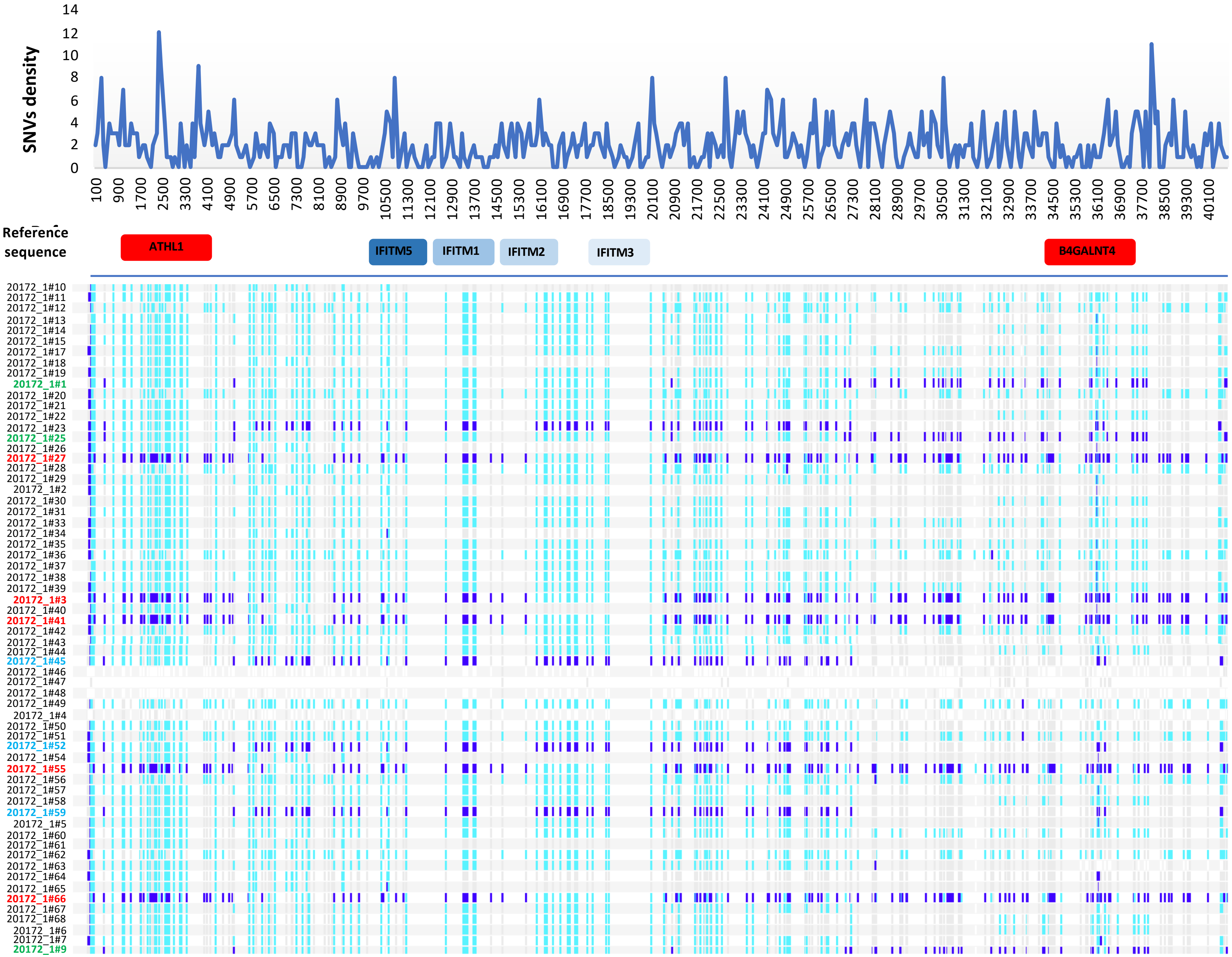

SNVs density
14
12
10
8
6
4
2
0
100
900
1700
2500
3300
4100
4900
5700
6500
7300
8100
8900
9700
10500
11300
12100
12900
13700
14500
15300
16100
16900
17700
18500
19300
20100
20900
21700
22500
23300
24100
24900
25700
26500
27300
28100
28900
29700
30500
31300
32100
32900
33700
34500
35300
36100
36900
37700
38500
39300
40100
Reference sequence
ATHL1
IFITM5
IFITM1
IFITM2
IFITM3
B4GALNT4
20172_1#10
20172_1#11
20172_1#12
20172_1#13
20172_1#14
20172_1#15
20172_1#17
20172_1#18
20172_1#19
20172_1#1
20172_1#20
20172_1#21
20172_1#22
20172_1#23
20172_1#25
20172_1#26
20172_1#27
20172_1#28
20172_1#29
20172_1#2
20172_1#30
20172_1#31
20172_1#33
20172_1#34
20172_1#35
20172_1#36
20172_1#37
20172_1#38
20172_1#39
20172_1#3
20172_1#40
20172_1#41
20172_1#42
20172_1#43
20172_1#44
20172_1#45
20172_1#46
20172_1#47
20172_1#48
20172_1#49
20172_1#4
20172_1#50
20172_1#51
20172_1#52
20172_1#54
20172_1#55
20172_1#56
20172_1#57
20172_1#58
20172_1#59
20172_1#5
20172_1#60
20172_1#61
20172_1#62
20172_1#63
20172_1#64
20172_1#65
20172_1#66
20172_1#67
20172_1#68
20172_1#6
20172_1#7
20172_1#9

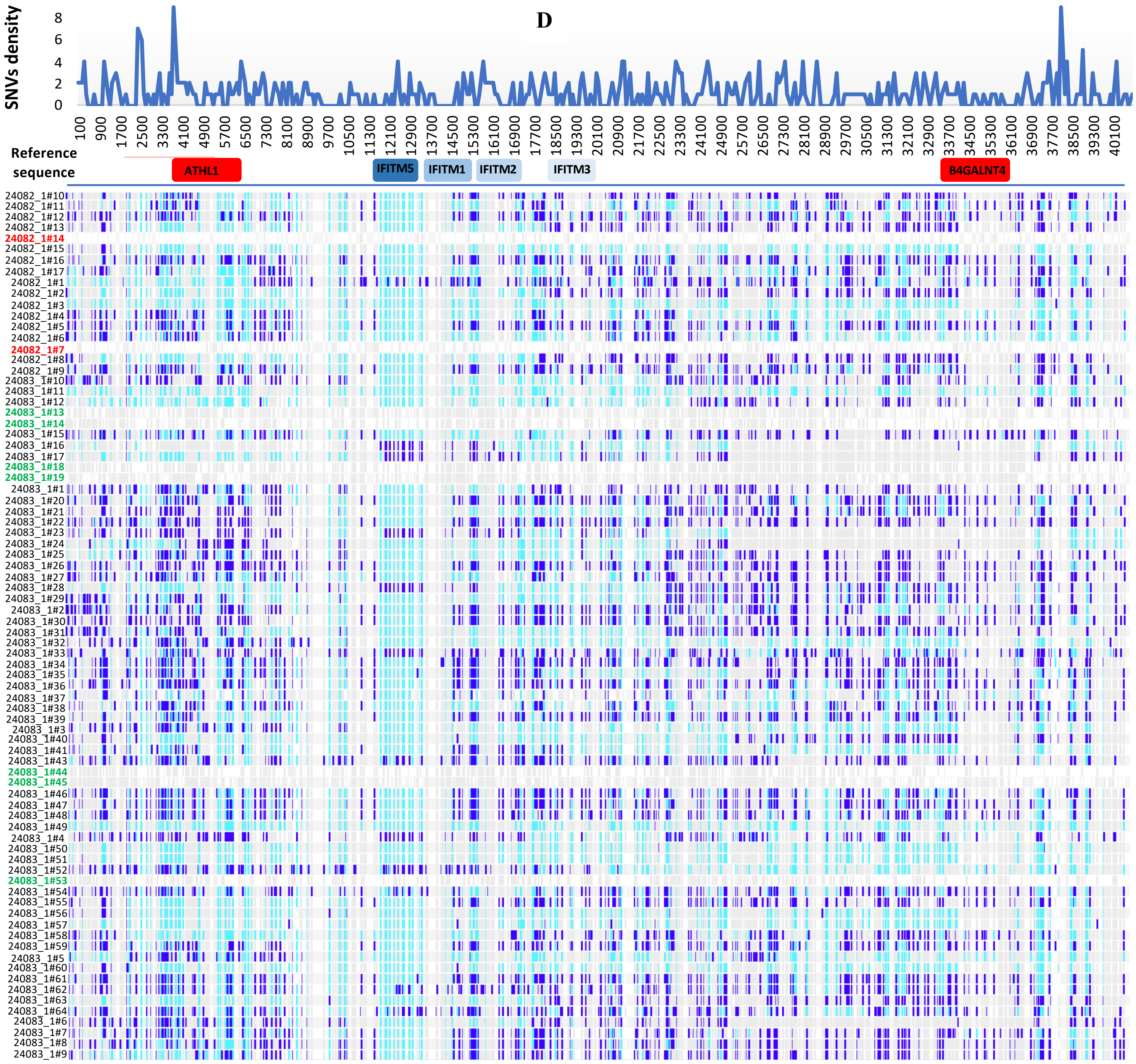

D
SNVs density
8
6
4
2
0
100
900
1700
2500
3300
4100
4900
5700
6500
7300
8100
8900
9700
10500
11300
12100
12900
13700
14500
15300
16100
16900
17700
18500
19300
20100
20900
21700
22500
23300
24100
24900
25700
26500
27300
28100
28900
29700
30500
31300
32100
32900
33700
34500
35300
36100
36900
37700
38500
39300
40100
Reference sequence
ATHL1
IFITM5
IFITM1
IFITM2
IFITM3
B4GALNT4
24082_1#10
24082_1#11
24082_1#12
24082_1#13
24082_1#14
24082_1#15
24082_1#16
24082_1#17
24082_1#1
24082_1#2
24082_1#3
24082_1#4
24082_1#5
24082_1#6
24082_1#7
24082_1#8
24082_1#9
24083_1#10
24083_1#11
24083_1#12
24083_1#13
24083_1#14
24083_1#15
24083_1#16
24083_1#17
24083_1#18
24083_1#19
24083_1#1
24083_1#20
24083_1#21
24083_1#22
24083_1#23
24083_1#24
24083_1#25
24083_1#26
24083_1#27
24083_1#28
24083_1#29
24083_1#2
24083_1#30
24083_1#31
24083_1#32
24083_1#33
24083_1#34
24083_1#35
24083_1#36
24083_1#37
24083_1#38
24083_1#39
24083_1#3
24083_1#40
24083_1#41
24083_1#43
24083_1#44
24083_1#45
24083_1#46
24083_1#47
24083_1#48
24083_1#49
24083_1#4
24083_1#50
24083_1#51
24083_1#52
24083_1#53
24083_1#54
24083_1#55
24083_1#56
24083_1#57
24083_1#58
24083_1#59
24083_1#5
24083_1#60
24083_1#61
24083_1#62
24083_1#63
24083_1#64
24083_1#6
24083_1#7
24083_1#8
24083_1#9
